# Physiological Roles of Apoptotic Cell Clearance: Beyond Immune Functions

**DOI:** 10.3390/life11111141

**Published:** 2021-10-26

**Authors:** Minjoo Han, Gyoungah Ryu, Seong-Ah Shin, Jangeun An, Huiji Kim, Daeho Park, Dae-Hee Lee, Chang Sup Lee

**Affiliations:** 1 College of Pharmacy and Research Institute of Pharmaceutical Sciences, Gyeongsang National University, Jinju 52828, Korea; dlsel79@gnu.ac.kr (M.H.); rga_97@gnu.ac.kr (G.R.); shinsaya@gnu.ac.kr (S.-A.S.); audtjraus98@gnu.ac.kr (J.A.); 2019080041@gnu.ac.kr (H.K.); 2 School of Life Sciences and Aging Research Institute, Gwangju Institute of Science and Technology, Gwangju 61005, Korea; daehopark@gist.ac.kr; 3 Department of Marine Food Science and Technology, Gangneung-Wonju National University, Gangneung 25456, Korea; neogene@gwnu.ac.kr

**Keywords:** apoptosis, apoptotic cell clearance, differentiation, development, inflammation, phagocyte

## Abstract

The clearance of apoptotic cells is known to be a critical step in maintaining tissue and organism homeostasis. This process is rapidly/promptly mediated by recruited or resident phagocytes. Phagocytes that engulf apoptotic cells have been closely linked to the release of anti-inflammatory cytokines to eliminate inflammatory responses. Defective clearance of apoptotic cells can cause severe inflammation and autoimmune responses due to secondary necrosis of apoptotic cells. Recently accumulated evidence indicates that apoptotic cells and their clearance have important physiological roles in addition to immune-related functions. Herein, we review the current understanding of the mechanisms and fundamental roles of apoptotic cell clearance and the beneficial roles of apoptotic cells in physiological processes such as differentiation and development.

## 1. Overview of Apoptotic Cell Clearance

There are various types of cell death such as apoptosis, pyroptosis, necroptosis, ferroptosis, and NETosis. In pyroptosis, pathologic stimuli and inflammatory caspases form pores in cell membranes [1]. Necroptosis is a programmed inflammatory cell death characterized by both apoptosis and necrosis [2]. Ferroptosis is an iron-dependent programmed cell death, in response to intracellular oxidative perturbations [3]. NETosis is a regulatory neutrophil cell death that occurs with the formation of neutrophil extracellular traps (NETs) [4]. These cell death types are also referred to as “regulated cell death’’ because various pathways define them at the molecular level [5]. In this review, we focused on apoptotic cell death and apoptotic cell clearance. Apoptosis is referred to as “programmed cell death”, which mediates normal development and tissue homeostasis in multicellular organisms [6,7,8]. Dysregulated apoptosis is known to cause a variety of pathological diseases, including cancer and neurodegenerative disorders [9,10,11]. Furthermore, cellular senescence is a state of irreversible cell cycle arrest exhibiting apoptosis resistance, and recent studies have found that it increases with aging and aging-related disease [12]. One form of cellular senescence called SASP (senescence-associated secretory phenotype) results in pathological outcomes [13]. During the past several decades, many critical results and scientific research data in the field of apoptosis have been obtained at the level of dying cells [6,9,14]. Currently, the removal of cell corpses after apoptosis in vivo is a major focus issue in this field [6].

Apoptotic cells arise under a variety of pathophysiological conditions, such as tissue remodeling during normal development, aging of cells, infection by viruses and bacteria, and damage by wounding [9]. Remarkably, the cell turnover rate in the human body is known to be approximately 1 million cells per second (>10^9^ per day) to maintain normal cellular and tissue homeostasis [15,16]. Although this indicates significant cell death per day, apoptotic cells are hardly detected in the body [15,16]. This implies that cell corpses are rapidly removed through a highly efficient mechanism for the clearance of apoptotic cells [15]. Professional phagocytes (macrophages, neutrophils, and immature dendritic cells) or non-professional (neighbor) phagocytes (fibroblasts, endothelial cells, and epithelial cells) have been implicated in apoptotic cell clearance with similar steps but different engulfment kinetics and capacity [6,17]. The main consequences of apoptotic cell clearance are “immunological silencing” through the physical removal of dying cells and “anti-inflammation” signals via the release of anti-inflammatory cytokines (transforming growth factor (TGF)-β and interleukin (IL)-10) in phagocytes [6,14,18]. Efficient and prompt removal of dying cells is very important for preventing apoptotic cells from undergoing secondary necrosis, which leads to the release of intracellular components via the disintegration of the plasma membrane [6,14,18]. The intracellular contents thus released may be toxic to neighboring cells and serve as immunogenic materials to induce strong inflammation [6,15,19]. Therefore, failure of apoptotic cell clearance can cause autoimmune diseases (autoimmunity, systemic lupus erythematosus, and rheumatoid arthritis) and immune-related disorders (chronic obstructive pulmonary disease, asthma, atherosclerosis, and cancer) [6,14,17,20,21,22,23,24,25,26,27,28,29]. Recent findings indicate that apoptotic cell clearance and dying cells themselves have unique roles in cellular differentiation and organ development, in addition to functioning as immunosuppressors [6]. Therefore, in this review, we focus on the current understanding of the special functions of apoptotic cell clearance under physiological conditions, as well as the mechanism of clearance of dying cells.

## 2. Mechanisms of Apoptotic Cell Clearance

**Figure 1:**
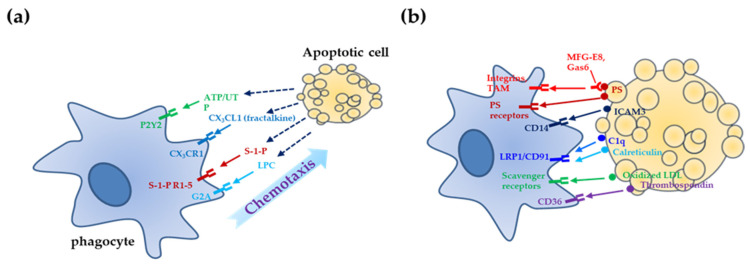

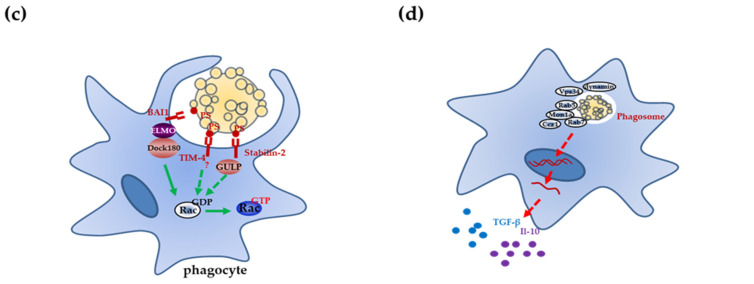
Sequential steps for apoptotic cell clearance. (**a**) The “find me” stage: apoptotic cells secrete “find me” signals (ATP/UTP, lysophosphatidylcholine (LPC), sphingosine-1-phosphate (S1P), and fractalkine (FKN/CX_3_CL1)) that can bind to receptors (P2Y2, G2A, S1P receptors, and CX_3_CR1) of phagocytes to induce chemotactic migration toward them. (**b**) The “eat me” stage: apoptotic cells expose “eat me” signals (phosphatidylserine (PS), intercellular adhesion molecule 3 (ICAM3), serum proteins (complement component 1q (C1q) and thrombospondin), intracellular proteins (calreticulin and annexin I), and oxidized low-density lipoprotein (LDL)) on their surface. Phagocytes recognize specific “eat me” signals with their receptors (phosphatidylserine (PS)/PS receptors (brain-specific angiogenesis inhibitor (BAI)-1, T cell immunoglobulin and mucin (TIM) family (TIM-1, 3, 4), and stabilin-2) or PS/soluble PS bridge molecules (milk fat globule-EGF factor 8 (MFG-E8) and growth arrest-specific 6 (Gas6)); these bridge molecules simultaneously bind to their receptors (integrin α_v_β_3/5_ and Tyro3-Axl-Mer tyrosine kinase (MerTK) family of receptors (TAM receptors), respectively) on phagocytes, and to calreticulin- LDL receptor–related protein-1 (LRP1)/CD91, C1q-LRP1/CD91, thrombospondin-CD36 (with integrin α_v_β_3/5_), ICAM3-CD14, and oxidized LDL-scavenger receptors. (**c**) The “engulfment/internalization” stage: recognition of “eat me” signals by phagocytic receptors induces cytoskeletal changes to engulf dying cells. There are two major pathways to activate Rac, which mediates the internalization of cell corpses (α_v_β_3/5_, TAM receptor, or BAI1 →CrkII-Dock180-ELMO → Rac, and LRP1/CD91 or stabilin-2 → GULP → Rac). (**d**) The “digestion and post-engulfment” stage: after internalizing dying cells, phagocytes degrade engulfed cells via a phagolysosomal pathway involving dynamin, Vps34, Rab5, Mon1a, Ccz1, and Rab7, and release anti-inflammatory cytokines (transforming growth factor (TGF)-β and interleukin (IL)-10) to perform the “immunological silencing” function.

Phagocytic processes to remove apoptotic cells can be categorized into four different levels (Figure 1): (a) the “find me” stage, wherein apoptotic cells release chemoattractants to recruit phagocytes; (b) the “eat me” stage, wherein phagocytes recognize “eat me” signals exposed on the surface of apoptotic cells; (c) the “engulfment/internalization” stage, wherein apoptotic corpses are internalized through cytoskeletal rearrangement of phagocytes; and (d) the “digestion and post-engulfment” stage, wherein internalized apoptotic cells are degraded through phagolysosomal processing in phagocytes, and phagocytes secrete anti-inflammatory cytokines [18,28,30]. 

At the “find me” stage, dying cells release “find me” signals such as nucleotides (ATP and UTP), lysophosphatidylcholine (LPC), sphingosine-1-phosphate (S1P), and fractalkine (CX_3_CL1). These molecules bind to their specific receptors (P2Y2, G2A, S1P receptors, and CX_3_CR1) on the phagocyte surface and induce the migration of phagocytes toward apoptotic cells [6,15,31]. Recently, it has been reported that traditional chemokines such as IL-8 and monocyte chemoattractant protein 1 (MCP-1) released by Fas/ cluster of differentiation (CD) 95-induced apoptotic cells can act as “find me” signals to promote the migration of phagocytes toward dying cells [32].

Phagocytes recognize the “eat me” signals on the surface of apoptotic cells to distinguish dying cells from normal cells in the vicinity [14,33]. These “eat me” signals are considered as markers of apoptotic cells [14,33,34,35]. They include exposure of phosphatidylserine (PS) on the outer leaflet of the plasma membrane, glycosylation of cell surface proteins, changes in cell surface charge, accumulation of serum proteins (complement component 1q (C1q) and thrombospondin), expression of intercellular adhesion molecule 3 (ICAM3), and exposure of intracellular proteins (calreticulin and annexin I) [14]. The most well-studied “eat me” signal is the exposure of PS [36,37]. In living cells, PS exclusively resides on the inner leaflet of the plasma membrane due to the action of ATP-dependent translocases in maintaining PS asymmetry [38]. When cells undergo apoptosis, dying cells show a dramatic exposure of PS on the outer leaflet of the plasma membrane [18]. Several studies have identified the molecules involved in PS exposure during apoptosis. Recently, Nagata et al. reported that transmembrane protein 16F (TMEM16F), a Ca^2+^-dependent phospholipid scramblase, is a key component for PS exposure on the surface of live cells (Ba/F3 cells) [39]. However, TMEM16F-dependent PS exposure on the surface of live cells did not cause engulfment by macrophages. Furthermore, they also identified the Xk-family protein (Xkr8) that mediates PS exposure on the apoptotic cell surface and promotes the removal of cell corpses [40]. Most recently, they identified that, in addition to scramblases (TMEM16F and Xkr8), adenosine triphosphatase type 11C (ATP11C) and cell division cycle protein 50A (CDC50A) are phospholipid flippases that can translocate PS from the outer membrane to the inner membrane, and these flippases are required for the localization of PS on the outer membrane of apoptotic cells [41,42]. The PS exposed on the apoptotic cell surface can be recognized by PS receptors (brain-specific angiogenesis inhibitor (BAI)-1, T cell immunoglobulin and mucin (TIM) family (TIM-1, 3, 4), and stabilin-2) or soluble PS bridge molecules (milk fat globule-EGF factor 8 (MFG-E8) and growth arrest-specific 6 (Gas6)) [6,15]. PS receptors on the surface of phagocytes can directly interact with PS on the apoptotic cell surface [6,33]. MFG-E8 and Gas6 bind to PS on the surface of apoptotic cells and simultaneously interact with their specific receptors (integrin α_v_β_3/5_ and Tyro3-Axl-Mer tyrosine kinase (MerTK) family of receptors (TAM receptors), respectively) on phagocytes [30,33]. In addition to PS and PS receptors, many molecules are known to be “eat me” signals, and their receptors include calreticulin-low density lipoprotein receptor-related protein 1 (LRP1/CD91), C1q-LRP1/CD91, thrombospondin-CD36 (with integrin α_v_β_3/5_), ICAM3-CD14, and oxidized low density lipoprotein (LDL)-scavenger receptors [18]. Furthermore, it has recently been suggested that several new members can serve as “eat me” signals or engulfment receptors. For example, Galectin-3 (Gal-3), an “eat me” signal, has been identified as a ligand for Mer, an engulfment receptor [43]. In addition, the transthyretin-like protein 52 (TTR-52) in *Caenorhabditis elegans* is known to be secreted into the extracellular region and function as a PS-binding molecule to mediate phagocytosis of apoptotic cells by the cell death abnormality protein 1 (CED-1) engulfment receptor [35]. Moreover, it has been reported that “triggering receptor expressed on myeloid cells (TREM)”-like protein 2 (TREML2/TLT2) can act as a phagocytic receptor to engulf apoptotic cells [44]. Furthermore, the receptor for advanced glycation end products, a new PS receptor, has been reported to bind to PS on the surface of dying cells and mediate the clearance of apoptotic cells [45]. Most recently, death domain 1α, a target gene of p53, was shown to play dual roles as an engulfment ligand or receptor that can mediate the engulfment of apoptotic cells through homophilic interaction [46]. Although a variety of “eat me” signals and engulfment receptors have been identified, further detailed characterizations including signaling mechanisms and in vivo relevance are still required to elucidate their functions.

Engagement of phagocytic receptors by “eat me” signals induces cytoskeletal reorganization to mediate the engulfment of apoptotic cells. It is known that the small GTPase Rac, a key mediator of actin-cytoskeletal dynamics, can be activated through two major signaling pathways for the internalization of cell corpses. Firstly, integrin α_v_β_3/5_, TAM receptor, and BAI1 can activate Rac to induce engulfment using the CrkII-Dock180-ELMO pathway [47,48,49]. Secondly, LRP1/CD91 and stabilin-2 can stimulate Rac activation through GULP [50,51,52]. The downstream signaling mediator of TIM-4 has not been identified. Moreover, TIM-4 has been suggested to be a “tethering receptor” that cannot mediate direct downstream signaling for the engulfment of apoptotic cells [53,54]. Recently, other signaling pathways in addition to the two main engulfment pathways have been suggested as independent pathways. ABL-1 and ABI-1 have been reported to be involved in engulfment via a pathway independent from the CrkII-Dock180-ELMO and GULP pathways [55]. It is also known that G-protein-coupled receptor kinase 6 (GRK6) and GRK-interacting ADP ribosylation factor GTPase-activating protein 1 (GIT1) can cooperatively activate Rac, which induces the engulfment of dying cells through a pathway that is distinct from the two main pathways [56]. These examples show that extensive investigations to identify and establish new signaling pathways are required to broaden the understanding of engulfment processes.

After internalization, engulfed corpses are further processed through the phagolysosomal pathway and digested into amino acids, nucleotides, and phospholipids. The sequential steps of phagosome maturation have common endocytic machinery as well as unique molecules, including dynamin, Vps34, Rab5, Mon1a, Ccz1, and Rab7 [57]. In addition to physical removal to prevent apoptotic cells from undergoing secondary necrosis, which leads to inflammatory responses, phagocytes can release anti-inflammatory cytokines (TGF-β and IL-10) as one of the main consequences of apoptotic cell clearance to achieve “immunological silencing” [6,30,34,58]. Four different stages of apoptotic cell clearance are highly regulated to achieve efficient and prompt clearance of such cells, and these can also be closely linked to one another. In particular, it has been suggested that the “find me” signal from dying cells can upregulate the engulfment receptors and/or engulfment-related machinery in phagocytes, as well as induce the recruitment of phagocytes [31]. In addition, sequential downstream events after engulfment have been known to affect the engulfment of apoptotic cells by phagocytes. For example, knockout (KO) studies of liver-X receptor (LXR) αβ and peroxisome proliferator-activated receptor (PPAR) γ/δ, which are the major players in lipid metabolism, have shown impaired apoptotic cell clearance [59,60,61]. The engulfment of apoptotic cells activates PPARs and LXR. These transcription factors then upregulate phagocytic receptors (MerTK and CD36) and opsonins (C1qb and MFG-E8) in phagocytes [62]. Moreover, Park et al. reported that uncoupling protein (UCP2) knockdown in phagocytes induced uncoupling between energy generation and nutrient digestion of apoptotic corpses in phagocytes, which led to the reduction of continued engulfment of apoptotic cells by phagocytes [63]. As a proton transporter, UCP2 induces the loss of the proton gradient across the inner mitochondrial membrane, eventually leading to a reduction in mitochondrial membrane potential without ATP generation. Therefore, through controlling the engulfment capacity of phagocytes, UCP2 regulation of mitochondrial membrane potential affects continued clearance. Therefore, crosstalk and internetworking among each stage is possible. However, ideas and experiments to prove these notions remain challenging.

## 3. Apoptotic Cell Clearance and Differentiation

**Figure 2:**
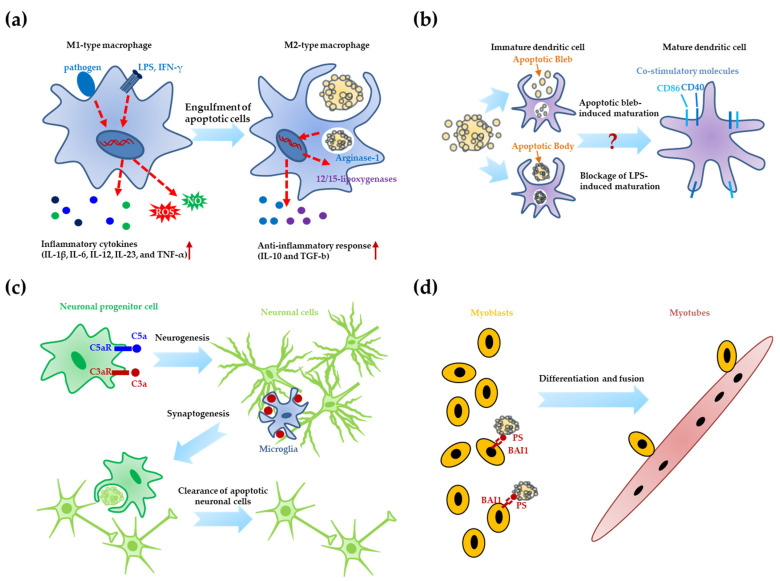
Roles of apoptotic cells in cellular differentiation. (**a**) M1-type macrophages that secrete inflammatory cytokines (IL-1β, IL-6, IL-12, IL-23, and tumor necrosis factor (TNF)-α), reactive oxygen species (ROS), and nitric oxide (NO) act as key immune effectors against intracellular microbes and clear affected apoptotic cells. After engulfing apoptotic cells, M1-type macrophages exhibit properties of M2-type macrophages (expression of arginase-1 and 12/15-lipoxygenases, and anti-inflammatory response (high IL-10 and TGF-β levels) involved in wound healing, tissue repair, and resolving of inflammation). (**b**) Engulfment of apoptotic blebs by immature dendritic cells can induce maturation of dendritic cells (increase in levels of co-stimulatory molecules CD40 and CD86) to activate T cells. Moreover, clearance of apoptotic cells by immature dendritic cells can block lipopolysaccharide (LPS)-induced maturation of dendritic cells. (**c**) Activation of complement receptors (C3a receptor (C3aR) and C5a receptor (C5aR)) on neuronal progenitor cells induces their differentiation and maturation in the central nervous system (CNS). Synaptic pruning in the postnatal retinogeniculate system can be induced by engulfment of developing presynaptic elements through complement receptor 3 (CR3-the receptor of iC3b, ICAM1, and fibrinogen) on microglia. Furthermore, removal of apoptotic neuronal cells by neuronal progenitor cells is critical in adult neurogenesis. (**d**) PS on apoptotic myoblast cells can be recognized by BAI1 (PS receptor) on neighboring myoblasts. Eventually, this mediates multinucleated myotube generation through muscle fusion/differentiation.

As mentioned above, clearance of dying cells can mediate not only immunologically silent events but also other special functions, including cellular differentiation and organ development. In this section, we discuss the effects of apoptotic cells on the differentiation of immune cells, neural cells, and muscle cells (Figure 2).

### 3.1. Differentiation of Immune Cells

M1 and M2 macrophages are differentiated from monocytes based on their exposure to mediators and environments [64,65]. Classically activated (M1) macrophages act as immune effectors against intracellular microbes and mediate the clearance of affected cells [64,65]. They release high levels of inflammatory cytokines (IL-1β, IL-6, IL-12, IL-23, and tumor necrosis factor (TNF)-α), reactive oxygen species (ROS), and nitric oxide (NO) [58,66,67,68]. However, alternatively activated (M2) macrophages secrete low levels of inflammatory cytokines and high levels of an anti-inflammatory cytokine (IL-10); they also express high levels of arginase-1 to interrupt NO generation and scavenger receptors [58,66,67,68,69,70]. M2 macrophages are known to mediate immune responses against extracellular parasites, wound healing, tissue repair, and resolving of inflammation [71,72,73,74]. Several reports suggest that after M1-type macrophages engulf apoptotic leukocytes, they exhibit features of M2-type macrophages, such as expression of arginase-1 and 12/15-lipoxygenases, and anti-inflammatory responses (low IL-12 and high IL-10 expression) [67]. Recently, CD36 and platelet-activating factor (PAF) receptor, which are implicated in apoptotic cell clearance, have been reported to be required for the characteristics of M2-type macrophages [75]. In addition, they have been reported to have a high capacity for apoptotic cell clearance [58]. Furthermore, it has been suggested that after M2-type macrophages are saturated with respect to the ability of clearing dying cells, they can be further changed into new types of macrophages (referred to as resolution-promoting macrophages), which might have anti-fibrotic and immune regulatory functions [58]. There have been several recent reports on the effects of engulfment of apoptotic bodies/cells on the maturation of dendritic cells, a type of professional phagocytes [76,77,78]. Fransen et al. reported that engulfment of apoptotic blebs, but not apoptotic cell bodies, induced dendritic cell maturation (increase in levels of co-stimulatory molecules CD40 and CD86), and the matured dendritic cells activated T cells to induce Th1/Th17 responses that mediated the induction of interferon (IFN)-γ and IL-17 [77,78]. However, Stuart et al. reported that the ingestion of apoptotic cells by immature dendritic cells blocked lipopolysaccharide (LPS)-mediated upregulation of CD86 expression in dendritic cells [76]. This result indicates that apoptotic cell clearance can mediate inhibitory effects on LPS-induced dendritic cell maturation. In the two studies mentioned above, apoptotic cell clearance played contradictory roles in dendritic cell maturation. However, each study used different apoptotic blebs/bodies/cells induced by different methods and cells. Therefore, the precise functions of apoptotic blebs, bodies, and cells in the maturation of dendritic cells need to be examined closely under well-defined conditions. Many studies have focused on the function of Mer tyrosine kinase (MerTK) as an engulfment receptor in apoptotic cell clearance. Research using knockout mice indicated that MerTK is implicated in T-cell-dependent B-cell differentiation, as its KO resulted reduced T-cell activation and B-cell differentiation [79]. However, the study did not clarify the exact mechanisms of MerTK action on T-cell activation and B-cell differentiation. Therefore, although the clearance of apoptotic cells can affect the differentiation of professional phagocytes (macrophages and dendritic cells), it is still unclear whether the engulfment of apoptotic cells is critical for the differentiation. Therefore, further studies are required to reveal whether apoptotic cell clearance is necessary and sufficient for the differentiation of these cells.

### 3.2. Neurogenesis

During neuronal development, many neuronal cells die, and the remaining surviving cells form neural circuits in the brain [80]. Complement proteins are known not only as mediators of immune responses via accumulation on the surface of apoptotic cells (“eat me” signal) but also as players with novel functions in tissue proliferation and regeneration [81]. In particular, it has been reported that complement factors (C3a and C5a) can mediate neurogenesis and synaptogenesis in the CNS and can directly act on neuronal progenitor cells to induce differentiation and maturation [82]. Furthermore, the engulfment of developing synapses using CR3 (the receptor of iC3b, ICAM1, and fibrinogen) on microglia has been recently reported to mediate synaptic pruning in the postnatal retinogeniculate system [82]. In addition, this report suggested that presynaptic elements engulfed by microglia were healthy and intact, but not apoptotic [82]. However, the functions of complement factors involved in neurogenesis and synaptogenesis remain elusive. Recently, engulfment of apoptotic cells through autophagy has been shown to be involved in the early stages of retinal development to develop the retinal neuroepithelium and inner ear development to remove neuroepithelial dying cells [83,84]. In addition, during the development of dorsal root ganglia (DRG) neurons, glial precursors play a key role in engulfment of apoptotic DRG neurons using Jedi-1 and multiple EGF like domains 10 (MEGF10) [85]. Furthermore, it has been reported that doublecortin (DCX)-positive neuronal progenitor cells can serve as phagocytes to engulf apoptotic neuronal cells in the adult brain, and their apoptotic cell clearance is critical to mediating adult neurogenesis [86]. Most recently, DCX-low human neural precursor cells and DCX-high neuroblasts have also been reported to be involved in apoptotic cell clearance via P2X7 during early neurogenesis [87]. Currently, there is not enough knowledge to fully explain the effects of cell clearance on neurogenesis, and there are many unanswered questions on the CNS, as follows: Which cells (neighbor cells or microglia/astrocytes) are involved in specific cell clearance in the CNS? Which “find me”, “eat me”, and phagocytic receptors contribute to engulfment activity for neurogenesis? Which mechanisms of cell clearance are important for neuronal development?

### 3.3. Muscle Differentiation

In skeletal muscle, mononucleated myoblasts turn into multinucleated myotubes through cell fusion/differentiation, which is a critical process for muscle development, regeneration, and repair [88,89]. Differentiation includes many different processes such as migration, cell-to-cell contact, fusion, and growth [90,91]. It has been reported that transient PS exposure (“eat me” signal, a unique feature of apoptotic cells) was detected on mouse embryonic myotubes at E13, and that PS exposure on the muscle cell surface is required for the fusion of myoblasts (muscle progenitor cells) into myotubes [92]. Furthermore, several studies have reported that PS was reversibly exposed on activated mast cell membranes after antigenic stimuli [93,94], although this exposure was not related to apoptosis. In addition, clusters of PS exposure are known to be localized in cell-to cell contact [95]. There are contradictory results regarding whether PS exposure and muscle fusion are dependent on the apoptosis of myoblasts [92]. Van den Eijnde et al. reported that PS exposure and muscle fusion cannot be inhibited by a pan-caspase inhibitor, whereas Fernando et al. found that caspase-3 activity for apoptosis is critical for myotube formation and induction of muscle-specific proteins [92,96]. Recently, Hochreiter-Hufford et al. reported that apoptosis through a pan-caspase inhibitor, zVAD, is required for PS exposure and muscle fusion, and that apoptotic cells themselves can work as novel players to induce muscle fusion/differentiation [97]. Furthermore, BAI1, a PS receptor, has been shown to be a key mediator for recognizing apoptotic cells and mediating myotube generation. This observation has generated a novel concept that apoptotic cells can not only be removed quickly but can also contribute to specific functions such as differentiation [97]. Most recently, BAI3, a member of the BAI family, has also been implicated in the myoblast fusion process [98]. However, the following question remains: How does the PS-BAI1 (BAI3) axis mediate the fusion processes of healthy myoblasts without engulfment of apoptotic cells?

## 4. Apoptotic Cell Clearance and Organogenesis

**Figure 3:**
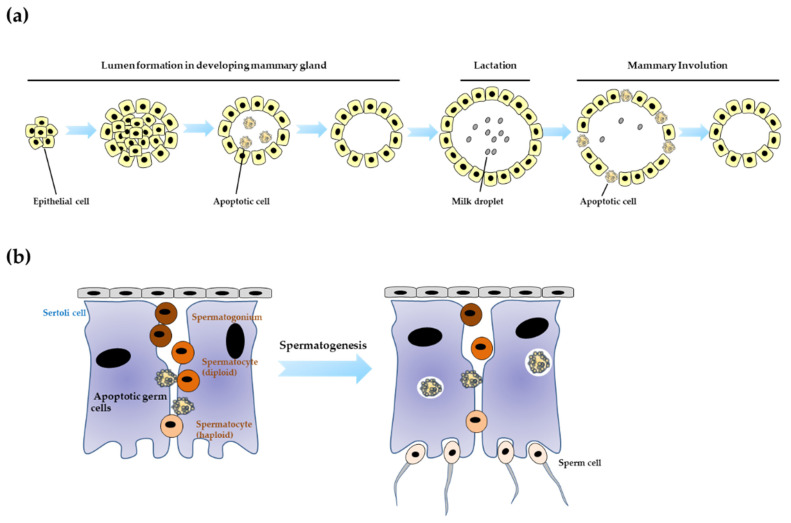
Roles of apoptotic cell clearance in organogenesis. (**a**) During mammary morphogenesis, removal of apoptotic mammary epithelial cells is essential for the formation of lumen. In addition, after weaning, residual milk and dying epithelial cells can be removed by engulfment of neighboring epithelial cells and macrophages to mediate mammary involution. (**b**) Spermatogenesis/sperm maturation is mediated by Sertoli cells that can control the maturation of germ cells to sperm cells via physical interaction. During spermatogenesis in testes, Sertoli cells act as phagocytic cells to remove defective sperm cells that have PS exposed. This process is critical for normal spermatogenesis.

Although there is substantial long-standing supportive evidence that clearance of dying cells during development essentially contributes to organogenesis/organ morphogenesis for specialized structures and functions [99,100], herein, we discuss only recent studies in the fields of mammary and testis development (Figure 3).

### 4.1. Mammary Development

In mammary morphogenesis, apoptosis of mammary epithelial cells and their clearance are well known to be critical for the formation of lumina (referred to as acini), which produce milk to feed newborns during lactation [100]. Recent advances in this field have focused on the relationship between the clearance of dying epithelial cells and mammary involution after weaning. Mammary involution is a step in which milk generation from the mammary gland is stopped and regression of the gland to the quiescent state is induced through removal of the remaining cells, apoptosis of the epithelial cells in the gland, clearance of dying cells, and regrowth of the interstitial adipocytes after weaning [101]. During the initial apoptosis of epithelial cells of mammary alveoli, the remaining neighbor epithelial cells that function as milk-secreting cells are changed to enable a role for phagocytes that mediate macropinocytosis to absorb milk and engulfment to remove apoptotic cells [101]. It has been suggested that this change can be induced upon contact with dying cells or by cytoskeletal reorganization of the surrounding epithelial cells during the extrusion of apoptotic cells. Recently, TGF-β signaling has been reported to induce junction reorganization between mammary epithelial cells for engulfment by epithelial cells [102]. At this stage, neighboring epithelial cells are known to induce many phagocytic activity-related genes such as those of receptors (LRP1/CD91 and CD47 (integrin-associated protein (IAP))), bridging molecules (Gas6 and protein S (ProS)), and lysosomal proteins (ATP6K and lysosomal-associated membrane protein 2 (LAMP2)). In addition, they can also express macrophage markers such as arginase-1 and inducible nitric oxide synthase (iNOS) during the early involution stages. Moreover, macrophages, which are professional phagocytes, are recruited to the glands around day 4 after weaning in the mouse system [101]. These observations strongly support the hypothesis that the engulfment activity of epithelial cells and macrophages contributes to the clearance of residual milk and dying epithelial cells during mammary involution. Furthermore, direct evidence and the in vivo relevance of the effects of apoptotic cell clearance on mammary involution have been revealed through KO mouse studies. Deficiency in MFG-E8, a bridging molecule that connects PS on apoptotic cells and engulfment receptors on phagocytes, impaired the redevelopment of the mammary gland and led to the development of periductal mastitis, which may have been caused by triggering the inflammatory process induced by secondary necrosis due to delayed clearance of dying cells [103]. In addition, loss of Disabled-2, an endocytic adaptor, impaired apoptotic cell clearance and delayed involution [104]. Furthermore, KO of Dock180 and Rac1, which are engulfment mediators, also resulted in the accumulation of apoptotic cells in the mammary gland at the late stage of involution, and delayed involution of the mammary glands [105,106]. Moreover, deficiency in autophagy-related protein 7 (ATG7) in mammary epithelial cells resulted in defective engulfment of apoptotic cells and a strong response to involution-related inflammation [107]. Although clearance of apoptotic epithelial cells has been suggested to be required for normal mammary involution based on direct and indirect evidence, many further investigations are needed to answer the following questions: What promotes the transition of epithelial cells into phagocytic cells post-weaning? Which cell types and receptors (molecules) are important for engulfment of dying epithelial cells during mammary involution?

### 4.2. Testis Development

During spermatogenesis in the testes, many germ cells undergo apoptotic cell death to obstruct the abnormal maturation of sperm with genetic or morphological defects [108,109]. Sperm maturation is closely linked to Sertoli cells located in the basal lamina of the seminiferous tubule [108]. Sertoli cells control the maturation of germ cells into spermatozoa through physical contact and by modulating the environment [108]. In particular, it is known that Sertoli cells carry out phagocytic clearance for apoptotic germ cells by recognizing PS exposure on the surface of dying cells and residual bodies to remove defective sperm cells during spermatogenesis [108]. Recently, several reports have shown the molecules involved in the phagocytic clearance of apoptotic sperm cells by Sertoli cells. Triple-null mice of TAM receptors (Tyro 3, Axl, and Mer) are known to show no mature sperm and a significant increase in the number of uncleared dying germ cells due to the impaired phagocytic activity of apoptotic differentiating germ cells among Sertoli cells [108,110,111,112,113].

In addition, Gas6, which links PS on apoptotic cells and TAM receptors on phagocytes, has been reported to be involved in the phagocytic activity of Sertoli cells to eliminate dying germ cell corpses [114]. Moreover, it has been suggested that the CD36 scavenger receptor can contribute to apoptotic germ cell clearance via its translocation to the plasma membranes of Sertoli cells [115]. Furthermore, injection of the BAI1-thrombospondin type 1 repeat (TSR) region (PS-binding region of BAI1) into testes increases the numbers of immature sperm and uncleared dying germ cells [116]. This indicates that BAI1 may also be involved in spermatogenesis and engulfment of apoptotic germ cells via the recognition of PS on the surface of dying cells [116]. In addition, Sertoli cell-specific deletion mice of Elmo1, which is known to be a direct downstream molecule of BAI1, also showed an increased number of uncleared dying germ cells and a significantly decreased number of mature sperm cells [116]. These reports indicate that there is a close relationship between efficient clearance of apoptotic germ cells and spermatogenesis to generate intact sperms [116]. Recently, the autophagy process has also been implicated in the ability of Sertoli cells to clear PS-exposed substrates [117]. Upon ingesting both PS-exposed legitimate and illegitimate substrate, Sertoli cells only degrade illegitimate substrates via autophagy pathways (MAP1LC3A-II/LC3-II clustering and SQSTM1/p62 degradation) [117]. Although there are several hypothetical scenarios in which apoptotic cell clearance may affect the interaction between Sertoli cells and germ cells, the integrity of the Sertoli cell barrier (tight junction), and the immune response after engulfment, the effect of phagocytic removal of dying germ cells on spermatogenesis is unknown [108].

## 5. Conclusions

Currently, it is known that “removal/clearance” issues are as essential as “making/synthesis” issues in a variety of pathophysiological processes. Apoptotic cell death and clearance of dying cells occur throughout the life of an organism (from the developmental stage to the end of life). Apoptotic cell clearance is known to have immunological silencing and anti-inflammatory effects via the efficient removal of apoptotic corpses by phagocytes to block secondary necrosis and the secretion of anti-inflammatory cytokines after the engulfment of dying cells by phagocytes [6,30,34,58]. However, recent accumulating findings on the roles of apoptotic cell clearance suggest a modification of these main concepts. In other words, the clearance of dying cells can contribute to physiological processes such as differentiation and development beyond immunosuppression-related pathways. Furthermore, attention must be paid to the role of apoptotic cells or clearance of dying cells as “new signal players,” which can directly cause differentiation and development. Currently, the functions of apoptotic cells and their clearance under a variety of pathophysiological conditions need to be thought of from a new point of view. In addition, tumor-associated macrophages (TAMs) are involved in activating immunosuppression and promoting tumor growth in the tumor microenvironment [118,119,120]. These TAMs are characteristic of M2 type macrophages producing anti-inflammatory cytokines. Therefore, phagocytes do not always function as the only cleaner of apoptotic cells. Inhibiting TAM activity is a potential therapeutic target for cancer prevention [121]. Another special issue is cellular cannibalism, which is defined as “cell-eat-cell” [122], with atypical phagocytosis by cancer cells being a clear example. This process is similar to phagocytosis by normal phagocytes, except that both living and dead cells are consumed [123]. These new discoveries broaden our understanding of cell removal, including living-cell clearance from atypical phagocytic cells in addition to apoptotic cell clearance from normal phagocytic cells.

However, there are many unanswered basic questions to be addressed before verifying this concept. For example, the reason why phagocytes recognize a variety of “eat me” signals, and the specific profiles of “eat me” signals and receptors in tissue-specific phagocytes (macrophages, dendritic cells, epithelial cells, and fibroblasts) are unknown. In addition, interrelationships and crosstalk among “eat me” signals, receptors, and/or phagocytes have not been determined. Additionally, downstream molecules and pathways of engulfment receptors need to be identified and characterized. Furthermore, investigating the function of apoptotic cells and their clearance in other pathophysiological processes, and identifying the “eat me” signals, engulfment receptors, and signaling pathways involved in differentiation and development processes will be the first step in understanding and verifying the new concept. Meta-data analysis and integration of new information and knowledge through systemic approaches such as the proteomic/genomic approach will be helpful in answering the above questions.

## References

[1] Jorgensen I., Miao E.A. (2015). Pyroptotic cell death defends against intracellular pathogens. Immunol. Rev..

[2] Nirmala J.G., Lopus M. (2020). Cell death mechanisms in eukaryotes. Cell Biol. Toxicol..

[3] Yang W.S., Stockwell B.R. (2016). Ferroptosis: Death by lipid peroxidation. Trends Cell Biol..

[4] Brostjan C., Oehler R. (2020). The role of neutrophil death in chronic inflammation and cancer. Cell Death Discov..

[5] Galluzzi L., Vitale I., Aaronson S.A., Abrams J.M., Adam D., Agostinis P., Alnemri E.S., Altucci L., Amelio I., Andrews D.W. (2018). Molecular mechanisms of cell death: Recommendations of the nomenclature committee on cell death 2018. Cell Death Differ..

[6] Gregory C.D., Pound J.D. (2011). Cell death in the neighbourhood: Direct microenvironmental effects of apoptosis in normal and neoplastic tissues. J. Pathol..

[7] Elmore S. (2007). Apoptosis: A review of programmed cell death. Toxicol. Pathol..

[8] Kumar S., Calianese D., Birge R.B. (2017). Efferocytosis of dying cells differentially modulate immunological outcomes in tumor microenvironment. Immunol. Rev..

[9] Singh R., Letai A., Sarosiek K. (2019). Regulation of apoptosis in health and disease: The balancing act of bcl-2 family proteins. Nat. Rev. Mol. Cell Biol..

[10] Wong R.S. (2011). Apoptosis in cancer: From pathogenesis to treatment. J. Exp. Clin. Cancer Res..

[11] Radi E., Formichi P., Battisti C., Federico A. (2014). Apoptosis and oxidative stress in neurodegenerative diseases. J. Alzheimer’s Dis..

[12] Payea M.J., Anerillas C., Tharakan R., Gorospe M. (2021). Translational control during cellular senescence. Mol. Cell. Biol..

[13] Di Micco R., Krizhanovsky V., Baker D., d’Adda di Fagagna F. (2021). Cellular senescence in ageing: From mechanisms to therapeutic opportunities. Nat. Rev. Mol. Cell Biol..

[14] Poon I.K., Lucas C.D., Rossi A.G., Ravichandran K.S. (2014). Apoptotic cell clearance: Basic biology and therapeutic potential. Nat. Rev. Immunol..

[15] Arandjelovic S., Ravichandran K.S. (2015). Phagocytosis of apoptotic cells in homeostasis. Nat. Immunol..

[16] Gregory C. (2009). Cell biology: Sent by the scent of death. Nature.

[17] Atkin-Smith G.K. (2021). Phagocytic clearance of apoptotic, necrotic, necroptotic and pyroptotic cells. Biochem. Soc. Trans..

[18] Ravichandran K.S., Lorenz U. (2007). Engulfment of apoptotic cells: Signals for a good meal. Nat. Rev. Immunol..

[19] Kourtzelis I., Hajishengallis G., Chavakis T. (2020). Phagocytosis of apoptotic cells in resolution of inflammation. Front. Immunol..

[20] Grabiec A.M., Hussell T. (2016). The role of airway macrophages in apoptotic cell clearance following acute and chronic lung inflammation. Semin. Immunopathol..

[21] Van Vre E.A., Ait-Oufella H., Tedgui A., Mallat Z. (2012). Apoptotic cell death and efferocytosis in atherosclerosis. Arterioscler. Thromb. Vasc. Biol..

[22] Schrijvers D.M., de Meyer G.R., Kockx M.M., Herman A.G., Martinet W. (2005). Phagocytosis of apoptotic cells by macrophages is impaired in atherosclerosis. Arterioscler. Thromb. Vasc. Biol..

[23] Thorp E., Cui D., Schrijvers D.M., Kuriakose G., Tabas I. (2008). Mertk receptor mutation reduces efferocytosis efficiency and promotes apoptotic cell accumulation and plaque necrosis in atherosclerotic lesions of apoe^−/−^ mice. Arterioscler. Thromb. Vasc. Biol..

[24] Cohen P.L., Caricchio R., Abraham V., Camenisch T.D., Jennette J.C., Roubey R.A., Earp H.S., Matsushima G., Reap E.A. (2002). Delayed apoptotic cell clearance and lupus-like autoimmunity in mice lacking the c-mer membrane tyrosine kinase. J. Exp. Med..

[25] Hanayama R., Tanaka M., Miyasaka K., Aozasa K., Koike M., Uchiyama Y., Nagata S. (2004). Autoimmune disease and impaired uptake of apoptotic cells in mfg-e8-deficient mice. Science.

[26] Tas S.W., Quartier P., Botto M., Fossati-Jimack L. (2006). Macrophages from patients with sle and rheumatoid arthritis have defective adhesion in vitro, while only sle macrophages have impaired uptake of apoptotic cells. Ann. Rheum. Dis..

[27] Grabiec A.M., Denny N., Doherty J.A., Happonen K.E., Hankinson J., Connolly E., Fife M.E., Fujimori T., Fujino N., Goenka A. (2017). Diminished airway macrophage expression of the axl receptor tyrosine kinase is associated with defective efferocytosis in asthma. J. Allergy Clin. Immunol..

[28] Gheibi Hayat S.M., Bianconi V., Pirro M., Sahebkar A. (2019). Efferocytosis: Molecular mechanisms and pathophysiological perspectives. Immunol. Cell Biol..

[29] Zhou Y., Yao Y., Deng Y., Shao A. (2020). Regulation of efferocytosis as a novel cancer therapy. Cell Commun. Signal..

[30] Ravichandran K.S. (2010). Find-me and eat-me signals in apoptotic cell clearance: Progress and conundrums. J. Exp. Med..

[31] Medina C.B., Ravichandran K.S. (2016). Do not let death do us part: ‘Find-me’ signals in communication between dying cells and the phagocytes. Cell Death Differ..

[32] Cullen S.P., Henry C.M., Kearney C.J., Logue S.E., Feoktistova M., Tynan G.A., Lavelle E.C., Leverkus M., Martin S.J. (2013). Fas/cd95-induced chemokines can serve as “find-me” signals for apoptotic cells. Mol. Cell.

[33] Nagata S. (2018). Apoptosis and clearance of apoptotic cells. Annu. Rev. Immunol..

[34] Nagata S., Hanayama R., Kawane K. (2010). Autoimmunity and the clearance of dead cells. Cell.

[35] Wang X., Li W., Zhao D., Liu B., Shi Y., Chen B., Yang H., Guo P., Geng X., Shang Z. (2010). Caenorhabditis elegans transthyretin-like protein ttr-52 mediates recognition of apoptotic cells by the ced-1 phagocyte receptor. Nat. Cell Biol..

[36] Wu Y., Tibrewal N., Birge R.B. (2006). Phosphatidylserine recognition by phagocytes: A view to a kill. Trends Cell Biol..

[37] Kenis H., van Genderen H., Bennaghmouch A., Rinia H.A., Frederik P., Narula J., Hofstra L., Reutelingsperger C.P. (2004). Cell surface-expressed phosphatidylserine and annexin a5 open a novel portal of cell entry. J. Biol. Chem..

[38] Cory S. (2018). Phosphatidylserine hide-and-seek. Proc. Natl. Acad. Sci. USA.

[39] Suzuki J., Umeda M., Sims P.J., Nagata S. (2010). Calcium-dependent phospholipid scrambling by tmem16f. Nature.

[40] Suzuki J., Denning D.P., Imanishi E., Horvitz H.R., Nagata S. (2013). Xk-related protein 8 and ced-8 promote phosphatidylserine exposure in apoptotic cells. Science.

[41] Segawa K., Kurata S., Yanagihashi Y., Brummelkamp T.R., Matsuda F., Nagata S. (2014). Caspase-mediated cleavage of phospholipid flippase for apoptotic phosphatidylserine exposure. Science.

[42] Suzuki J., Nagata S. (2014). Phospholipid scrambling on the plasma membrane. Methods Enzymol..

[43] Caberoy N.B., Alvarado G., Bigcas J.L., Li W. (2012). Galectin-3 is a new mertk-specific eat-me signal. J. Cell. Physiol..

[44] De Freitas A., Banerjee S., Xie N., Cui H., Davis K.I., Friggeri A., Fu M., Abraham E., Liu G. (2012). Identification of tlt2 as an engulfment receptor for apoptotic cells. J. Immunol..

[45] Armstrong A., Ravichandran K.S. (2011). Phosphatidylserine receptors: What is the new rage?. EMBO Rep..

[46] Yoon K.W., Byun S., Kwon E., Hwang S.Y., Chu K., Hiraki M., Jo S.H., Weins A., Hakroush S., Cebulla A. (2015). Cell death. Control of signaling-mediated clearance of apoptotic cells by the tumor suppressor p53. Science.

[47] Albert M.L., Kim J.I., Birge R.B. (2000). Alphavbeta5 integrin recruits the crkii-dock180-rac1 complex for phagocytosis of apoptotic cells. Nat. Cell Biol..

[48] Park D., Tosello-Trampont A.C., Elliott M.R., Lu M., Haney L.B., Ma Z., Klibanov A.L., Mandell J.W., Ravichandran K.S. (2007). Bai1 is an engulfment receptor for apoptotic cells upstream of the elmo/dock180/rac module. Nature.

[49] Abu-Thuraia A., Gauthier R., Chidiac R., Fukui Y., Screaton R.A., Gratton J.P., Cote J.F. (2015). Axl phosphorylates elmo scaffold proteins to promote rac activation and cell invasion. Mol. Cell. Biol..

[50] Park S.Y., Kang K.B., Thapa N., Kim S.Y., Lee S.J., Kim I.S. (2008). Requirement of adaptor protein gulp during stabilin-2-mediated cell corpse engulfment. J. Biol. Chem..

[51] Park S.Y., Kim I.S. (2017). Engulfment signals and the phagocytic machinery for apoptotic cell clearance. Exp. Mol. Med..

[52] Kinchen J.M., Ravichandran K.S. (2007). Journey to the grave: Signaling events regulating removal of apoptotic cells. J. Cell Sci..

[53] Wong K., Valdez P.A., Tan C., Yeh S., Hongo J.A., Ouyang W. (2010). Phosphatidylserine receptor tim-4 is essential for the maintenance of the homeostatic state of resident peritoneal macrophages. Proc. Natl. Acad. Sci. USA.

[54] Park D., Hochreiter-Hufford A., Ravichandran K.S. (2009). The phosphatidylserine receptor tim-4 does not mediate direct signaling. Curr. Biol..

[55] Hurwitz M.E., Vanderzalm P.J., Bloom L., Goldman J., Garriga G., Horvitz H.R. (2009). Abl kinase inhibits the engulfment of apoptotic [corrected] cells in caenorhabditis elegans. PLoS Biol..

[56] Nakaya M., Tajima M., Kosako H., Nakaya T., Hashimoto A., Watari K., Nishihara H., Ohba M., Komiya S., Tani N. (2013). Grk6 deficiency in mice causes autoimmune disease due to impaired apoptotic cell clearance. Nat. Commun..

[57] Kinchen J.M., Ravichandran K.S. (2008). Phagosome maturation: Going through the acid test. Nat. Rev. Mol. Cell Biol..

[58] Korns D., Frasch S.C., Fernandez-Boyanapalli R., Henson P.M., Bratton D.L. (2011). Modulation of macrophage efferocytosis in inflammation. Front. Immunol..

[59] Mukundan L., Odegaard J.I., Morel C.R., Heredia J.E., Mwangi J.W., Ricardo-Gonzalez R.R., Goh Y.P., Eagle A.R., Dunn S.E., Awakuni J.U. (2009). Ppar-delta senses and orchestrates clearance of apoptotic cells to promote tolerance. Nat. Med..

[60] Majai G., Sarang Z., Csomos K., Zahuczky G., Fesus L. (2007). Ppargamma-dependent regulation of human macrophages in phagocytosis of apoptotic cells. Eur. J. Immunol..

[61] Mota A.C., Dominguez M., Weigert A., Snodgrass R.G., Namgaladze D., Brune B. (2021). Lysosome-dependent lxr and ppardelta activation upon efferocytosis in human macrophages. Front. Immunol..

[62] Han C.Z., Ravichandran K.S. (2011). Metabolic connections during apoptotic cell engulfment. Cell.

[63] Park D., Han C.Z., Elliott M.R., Kinchen J.M., Trampont P.C., Das S., Collins S., Lysiak J.J., Hoehn K.L., Ravichandran K.S. (2011). Continued clearance of apoptotic cells critically depends on the phagocyte ucp2 protein. Nature.

[64] Yunna C., Mengru H., Lei W., Weidong C. (2020). Macrophage m1/m2 polarization. Eur. J. Pharmacol..

[65] Orecchioni M., Ghosheh Y., Pramod A.B., Ley K. (2019). Macrophage polarization: Different gene signatures in m1(lps+) vs. Classically and m2(lps−) vs. Alternatively activated macrophages. Front. Immunol..

[66] Filardy A.A., Pires D.R., Nunes M.P., Takiya C.M., Freire-de-Lima C.G., Ribeiro-Gomes F.L., DosReis G.A. (2010). Proinflammatory clearance of apoptotic neutrophils induces an il-12(low)il-10(high) regulatory phenotype in macrophages. J. Immunol..

[67] Schif-Zuck S., Gross N., Assi S., Rostoker R., Serhan C.N., Ariel A. (2011). Saturated-efferocytosis generates pro-resolving cd11b low macrophages: Modulation by resolvins and glucocorticoids. Eur. J. Immunol..

[68] Mantovani A., Sica A., Locati M. (2005). Macrophage polarization comes of age. Immunity.

[69] Murray P.J., Allen J.E., Biswas S.K., Fisher E.A., Gilroy D.W., Goerdt S., Gordon S., Hamilton J.A., Ivashkiv L.B., Lawrence T. (2014). Macrophage activation and polarization: Nomenclature and experimental guidelines. Immunity.

[70] Ivashkiv L.B. (2013). Epigenetic regulation of macrophage polarization and function. Trends Immunol..

[71] Satoh T., Takeuchi O., Vandenbon A., Yasuda K., Tanaka Y., Kumagai Y., Miyake T., Matsushita K., Okazaki T., Saitoh T. (2010). The jmjd3-irf4 axis regulates m2 macrophage polarization and host responses against helminth infection. Nat. Immunol..

[72] Sica A., Mantovani A. (2012). Macrophage plasticity and polarization: In vivo veritas. J. Clin. Investig..

[73] Ferrante C.J., Leibovich S.J. (2012). Regulation of macrophage polarization and wound healing. Adv. Wound Care.

[74] Pollard J.W. (2009). Trophic macrophages in development and disease. Nat. Rev. Immunol..

[75] Ferracini M., Rios F.J., Pecenin M., Jancar S. (2013). Clearance of apoptotic cells by macrophages induces regulatory phenotype and involves stimulation of cd36 and platelet-activating factor receptor. Mediat. Inflamm..

[76] Stuart L.M., Lucas M., Simpson C., Lamb J., Savill J., Lacy-Hulbert A. (2002). Inhibitory effects of apoptotic cell ingestion upon endotoxin-driven myeloid dendritic cell maturation. J. Immunol..

[77] Fransen J.H., Hilbrands L.B., Ruben J., Stoffels M., Adema G.J., van der Vlag J., Berden J.H. (2009). Mouse dendritic cells matured by ingestion of apoptotic blebs induce t cells to produce interleukin-17. Arthritis Rheum..

[78] Fransen J.H., van der Vlag J., Ruben J., Adema G.J., Berden J.H., Hilbrands L.B. (2010). The role of dendritic cells in the pathogenesis of systemic lupus erythematosus. Arthritis Res. Ther..

[79] Shao W.H., Zhen Y., Finkelman F.D., Cohen P.L. (2014). The mertk receptor tyrosine kinase promotes t-b interaction stimulated by igd b-cell receptor cross-linking. J. Autoimmun..

[80] Inokuchi K. (2011). Adult neurogenesis and modulation of neural circuit function. Curr. Opin. Neurobiol..

[81] Kouser L., Madhukaran S.P., Shastri A., Saraon A., Ferluga J., Al-Mozaini M., Kishore U. (2015). Emerging and novel functions of complement protein c1q. Front. Immunol..

[82] Crehan H., Hardy J., Pocock J. (2012). Microglia, Alzheimer’s disease, and complement. Int. J. Alzheimer’s Dis..

[83] Aburto M.R., Sanchez-Calderon H., Hurle J.M., Varela-Nieto I., Magarinos M. (2012). Early otic development depends on autophagy for apoptotic cell clearance and neural differentiation. Cell Death Dis..

[84] Mellen M.A., de la Rosa E.J., Boya P. (2008). The autophagic machinery is necessary for removal of cell corpses from the developing retinal neuroepithelium. Cell Death Differ..

[85] Wu H.H., Bellmunt E., Scheib J.L., Venegas V., Burkert C., Reichardt L.F., Zhou Z., Farinas I., Carter B.D. (2009). Glial precursors clear sensory neuron corpses during development via jedi-1, an engulfment receptor. Nat. Neurosci..

[86] Lu Z., Elliott M.R., Chen Y., Walsh J.T., Klibanov A.L., Ravichandran K.S., Kipnis J. (2011). Phagocytic activity of neuronal progenitors regulates adult neurogenesis. Nat. Cell Biol..

[87] Lovelace M.D., Gu B.J., Eamegdool S.S., Weible M.W., Wiley J.S., Allen D.G., Chan-Ling T. (2015). P2x7 receptors mediate innate phagocytosis by human neural precursor cells and neuroblasts. Stem Cells.

[88] Endo T. (2015). Molecular mechanisms of skeletal muscle development, regeneration, and osteogenic conversion. Bone.

[89] Tu M.K., Levin J.B., Hamilton A.M., Borodinsky L.N. (2016). Calcium signaling in skeletal muscle development, maintenance and regeneration. Cell Calcium.

[90] Abmayr S.M., Pavlath G.K. (2012). Myoblast fusion: Lessons from flies and mice. Development.

[91] Yu S.F., Baylies M.K. (2013). Cell biology: Death brings new life to muscle. Nature.

[92] Van den Eijnde S.M., van den Hoff M.J., Reutelingsperger C.P., van Heerde W.L., Henfling M.E., Vermeij-Keers C., Schutte B., Borgers M., Ramaekers F.C. (2001). Transient expression of phosphatidylserine at cell-cell contact areas is required for myotube formation. J. Cell Sci..

[93] Martin S., Pombo I., Poncet P., David B., Arock M., Blank U. (2000). Immunologic stimulation of mast cells leads to the reversible exposure of phosphatidylserine in the absence of apoptosis. Int. Arch. Allergy Immunol..

[94] Rysavy N.M., Shimoda L.M., Dixon A.M., Speck M., Stokes A.J., Turner H., Umemoto E.Y. (2014). Beyond apoptosis: The mechanism and function of phosphatidylserine asymmetry in the membrane of activating mast cells. Bioarchitecture.

[95] Jeong J., Conboy I.M. (2011). Phosphatidylserine directly and positively regulates fusion of myoblasts into myotubes. Biochem. Biophys. Res. Commun..

[96] Fernando P., Kelly J.F., Balazsi K., Slack R.S., Megeney L.A. (2002). Caspase 3 activity is required for skeletal muscle differentiation. Proc. Natl. Acad. Sci. USA.

[97] Hochreiter-Hufford A.E., Lee C.S., Kinchen J.M., Sokolowski J.D., Arandjelovic S., Call J.A., Klibanov A.L., Yan Z., Mandell J.W., Ravichandran K.S. (2013). Phosphatidylserine receptor bai1 and apoptotic cells as new promoters of myoblast fusion. Nature.

[98] Hamoud N., Tran V., Croteau L.P., Kania A., Cote J.F. (2014). G-protein coupled receptor bai3 promotes myoblast fusion in vertebrates. Proc. Natl. Acad. Sci. USA.

[99] Erwig L.P., Henson P.M. (2007). Immunological consequences of apoptotic cell phagocytosis. Am. J. Pathol..

[100] Mailleux A.A., Overholtzer M., Brugge J.S. (2008). Lumen formation during mammary epithelial morphogenesis: Insights from in vitro and in vivo models. Cell Cycle.

[101] Monks J., Henson P.M. (2009). Differentiation of the mammary epithelial cell during involution: Implications for breast cancer. J. Mammary Gland Biol. Neoplasia.

[102] Fornetti J., Flanders K.C., Henson P.M., Tan A.C., Borges V.F., Schedin P. (2015). Mammary epithelial cell phagocytosis downstream of tgf-beta3 is characterized by adherens junction reorganization. Cell Death Differ..

[103] Hanayama R., Nagata S. (2005). Impaired involution of mammary glands in the absence of milk fat globule egf factor 8. Proc. Natl. Acad. Sci. USA.

[104] Tao W., Moore R., Smith E.R., Xu X.X. (2014). Hormonal induction and roles of disabled-2 in lactation and involution. PLoS ONE.

[105] Akhtar N., Li W., Mironov A., Streuli C.H. (2016). Rac1 controls both the secretory function of the mammary gland and its remodeling for successive gestations. Dev. Cell.

[106] Bagci H., Laurin M., Huber J., Muller W.J., Cote J.F. (2014). Impaired cell death and mammary gland involution in the absence of dock1 and rac1 signaling. Cell Death Dis..

[107] Teplova I., Lozy F., Price S., Singh S., Barnard N., Cardiff R.D., Birge R.B., Karantza V. (2013). Atg proteins mediate efferocytosis and suppress inflammation in mammary involution. Autophagy.

[108] Griswold M.D. (1998). The central role of sertoli cells in spermatogenesis. Semin. Cell Dev. Biol..

[109] Shukla K.K., Mahdi A.A., Rajender S. (2012). Apoptosis, Spermatogenesis and male infertility. Front. Biosci..

[110] Elliott M.R., Ravichandran K.S. (2010). Elmo1 signaling in apoptotic germ cell clearance and spermatogenesis. Ann. N. Y. Acad. Sci..

[111] Zhang Y., Li N., Chen Q., Yan K., Liu Z., Zhang X., Liu P., Chen Y., Han D. (2013). Breakdown of immune homeostasis in the testis of mice lacking tyro3, axl and mer receptor tyrosine kinases. Immunol. Cell Biol..

[112] Chen Y., Wang H., Qi N., Wu H., Xiong W., Ma J., Lu Q., Han D. (2009). Functions of tam rtks in regulating spermatogenesis and male fertility in mice. Reproduction.

[113] Lu Q., Gore M., Zhang Q., Camenisch T., Boast S., Casagranda F., Lai C., Skinner M.K., Klein R., Matsushima G.K. (1999). Tyro-3 family receptors are essential regulators of mammalian spermatogenesis. Nature.

[114] Xiong W., Chen Y., Wang H., Wang H., Wu H., Lu Q., Han D. (2008). Gas6 and the tyro 3 receptor tyrosine kinase subfamily regulate the phagocytic function of sertoli cells. Reproduction.

[115] Gillot I., Jehl-Pietri C., Gounon P., Luquet S., Rassoulzadegan M., Grimaldi P., Vidal F. (2005). Germ cells and fatty acids induce translocation of cd36 scavenger receptor to the plasma membrane of sertoli cells. J. Cell Sci..

[116] Elliott M.R., Zheng S., Park D., Woodson R.I., Reardon M.A., Juncadella I.J., Kinchen J.M., Zhang J., Lysiak J.J., Ravichandran K.S. (2010). Unexpected requirement for elmo1 in clearance of apoptotic germ cells in vivo. Nature.

[117] Yefimova M.G., Messaddeq N., Harnois T., Meunier A.C., Clarhaut J., Noblanc A., Weickert J.L., Cantereau A., Philippe M., Bourmeyster N. (2013). A chimerical phagocytosis model reveals the recruitment by sertoli cells of autophagy for the degradation of ingested illegitimate substrates. Autophagy.

[118] Shin S.A., Moon S.Y., Park D., Park J.B., Lee C.S. (2019). Apoptotic cell clearance in the tumor microenvironment: A potential cancer therapeutic target. Arch. Pharmacal Res..

[119] Pan Y., Yu Y., Wang X., Zhang T. (2020). Tumor-associated macrophages in tumor immunity. Front. Immunol..

[120] Cassetta L., Pollard J.W. (2020). Tumor-associated macrophages. Curr. Biol..

[121] Lin Y., Xu J., Lan H. (2019). Tumor-associated macrophages in tumor metastasis: Biological roles and clinical therapeutic applications. J. Hematol. Oncol..

[122] Kale A. (2015). Cellular cannibalism. J. Oral Maxillofac. Pathol..

[123] Lozupone F., Fais S. (2015). Cancer cell cannibalism: A primeval option to survive. Curr. Mol. Med..

